# Association of School Residential PM_2.5_ with Childhood High Blood Pressure: Results from an Observational Study in 6 Cities in China

**DOI:** 10.3390/ijerph16142515

**Published:** 2019-07-14

**Authors:** Xijie Wang, Zhiyong Zou, Bin Dong, Yanhui Dong, Yinghua Ma, Di Gao, Zhaogeng Yang, Shaowei Wu, Jun Ma

**Affiliations:** 1 Institute of Child and Adolescent Health & School of Public Health, Peking University Health Science Center, Beijing 100191, China; 2 Key laboratory of Reproductive Health, National Health Commission of the People’s Republic of China, Beijing 100191, China; 3 School of Public Health, Peking University, Beijing 100191, China; 4 Key Laboratory of Molecular Cardiovascular Sciences, Peking University, Ministry of Education, Beijing 100191, China

**Keywords:** air pollution, particulate matter, high blood pressure, children

## Abstract

Objective: To investigate the association of long-term PM_2.5_ exposure with blood pressure (BP) outcomes in children aged 6–18 years, and to examine the population attributable risk (PAR) of PM_2.5_ exposure. Methods: A total of 53,289 participants aged 6–18 years with full record of age, sex, BP, height, and local PM_2.5_ exposure from a cross-sectional survey conducted in 6 cities of China in 2013 were involved in the present study. PM_2.5_ data from 18 January 2013 to 31 December 2013 were obtained from the nearest environmental monitoring station for each selected school. Two-level linear and logistic regression models were used to evaluate the influence of PM_2.5_ on children’s BP, and PAR was calculated in each sex and age group. Results: Participants had a mean age of 10.8 (standard deviation: 3.4) years at enrollment, 51.7% of them were boys. U-shaped trends along with increased PM_2.5_ concentration were found for both systolic blood pressure (SBP) and diastolic blood pressure (DBP), with the thresholds of 57.8 and 65.0 μg/m^3^, respectively. Both increased annual mean of PM_2.5_ concentration and ratio of polluted days were associated with increased BP levels and high blood pressure (HBP), with effect estimates for BP ranging from 2.80 (95% CI: −0.51, 6.11) mmHg to 5.78 (95% CI: 2.32, 9.25) mmHg for SBP and from 0.77 (95% CI: −1.98, 3.52) mmHg to 2.66 (−0.35, 5.66) mmHg for DBP, and the odds ratios for HBP from 1.21 (0.43, 3.38) to 1.92 (0.65, 5.67) in the highest vs. the lowest quartiles. Overall, 1.16% of HBP in our participants could be attributed to increased annual mean of PM_2.5_ concentration, while 2.82% could be attributed to increased ratio of polluted days. These proportions increased with age. Conclusions: The association between long-term PM_2.5_ exposure and BP values appeared to be U-shaped in Chinese children aged 6–18 years, and increased PM_2.5_ exposure was associated with higher risk of HBP.

## 1. Introduction

Hypertension or high blood pressure (HBP) is one of the largest contributors to cardiovascular disease disability-adjusted life-years (DALYs) [1]. In China, 6.4% of school-aged children have HBP and a considerable part of them would develop hypertension in their adulthood [2,3]. During the past decades, the prevalence of HBP raised for approximately 50% in Chinese children and makes it a huge public health problem [2]. Apart from those well-studied factors like genetic factors [4] or changes of lifestyles and diet structure [5], this increasing trend may also be associated with environmental factors, especially ambient air pollution [6].

Ambient air pollution, especially represented by PM_2.5_ (fine particulate matter with an aerodynamic ≤2.5 μm), has become one of the major environmental health challenges globally [7,8] and causes considerable burden of disease [9,10,11]. Previous studies, either domestic [12,13] or abroad, [14,15,16,17,18] have found associations, most of which were positive, between air pollution (whether short-term or long-term) and BP in adults. However, only a few studies have investigated the above associations in adolescents, a rather more environmental-sensitive population, and the few results have been controversial. Results from the Prevention and Incidence of Asthma and Mite Allergy (PIAMA) birth cohort study in Netherland found that long-term exposure to PM_2.5_ absorbance was significantly associated with increased diastolic blood pressure (DBP) [19,20] with 0.83 (0.06 to 1.61) mmHg in highly-exposed children compared to 0.75 (−0.08 to 1.58) mmHg in less-exposed children. Sughis M and colleagues from Pakistan found that both systolic blood pressure (SBP) and DBP increased with increasing PM_2.5_ exposure [21], whereas results from The German Infant Nutritional Intervention plus environmental and genetic influences on allergy development study (GINIplus) and The Lifestyle-Related factors on the Immune System and the Development of Allergies in Childhood plus the influence of traffic emissions and genetics (LISAplus) studies, which were two birth cohorts from Germany, suggested that this association may be contributed by other environmental exposure [22]. Therefore, further studies with large sample sizes are still in need to be carried out to investigate the association between ambient PM_2.5_ and childhood BP.

In this study, we investigated the association between ambient PM_2.5_ and BP in Chinese children aged 6–18 years, and also calculated the population attributable risk (PAR) of childhood HBP due to ambient PM_2.5_, using data from a large school-based cross-sectional study conducted in 6 different provinces in China.

## 2. Materials and Methods

### 2.1. Study Cities Selection and Subject Recruitment

**Figure 1:**
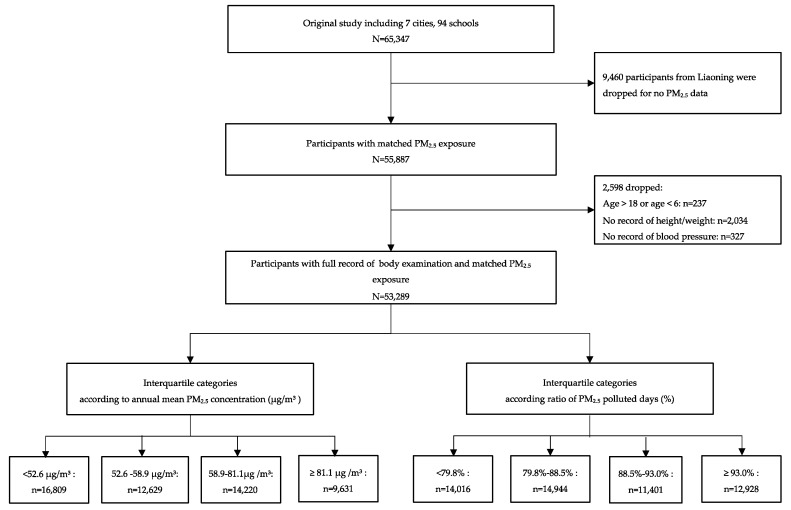
Diagram of subject recruitment.

The current study used data from a cross-sectional survey which was conducted in 7 provinces (Ningxia, Guangdong, Shanghai, Chongqing, Hunan, Tianjin, and Liaoning) in 2013. The sampling procedure of this study has been published in detail elsewhere [23]. Briefly, 65,347 participants from 94 schools in 7 cities out of the 7 provinces were randomly selected in the survey. For the present study, 9460 participants from 12 schools in Liaoning were dropped because local PM_2.5_ data were unavailable. Participants aged 6–18 years with full record of weight, height, and blood pressure measurement were involved in the present study; the subjects’ recruitment procedure is displayed in Figure 1. After this, 53,289 students aged 6–18 years with full record of PM_2.5_ exposure and health data from 82 schools were included in final data analysis. The original study had been approved by the Ethical Committee of Peking University (No. IRB0000105213034). All students participated and their parents have signed the informed consent.

### 2.2. Measurements of Physical Examinations

Height and weight of all participants were measured according to the study protocol [23]. Height was measured using the portable stadiometer (model TZG, China) to the nearest 0.1 cm, with the students standing straight barefoot. Weight was measured using lever-type weight scale (model RGT-140, China) to the nearest 0.1 kg, with students wearing light garments only. Both height and weight were measured twice and the mean values were recorded. Body mass index (BMI) was then calculated using the averaged weight (kg) divided by height (m) squared (kg/m^2^).

BP was measured using a mercury sphygmomanometer (model XJ11D, China) and a stethoscope (model TZ-1, China) from the right arm with an appropriate cuff. Students were instructed to be seated comfortably for at least 5 min prior to the first reading. SBP was determined by onset of the first Korotkoff sound and DBP was determined by the fifth Korotkoff sound. BP was measured twice with a one-minute interval. The averages of SBP and DBP were calculated from the two measurements. HBP was defined as average systolic or diastolic BPs ≥ 95th percentile for gender, age, and height, according to the fourth report on the diagnosis, evaluation, and treatment of HBP in children and adolescents [24].

### 2.3. Covariates

Information on daily consumption of fruits, daily consumption of vegetables, smoking exposure, and family history of hypertension was obtained from questionnaires from both students and their parents. The questionnaires of this survey were developed with reference to the Chinese National Survey on Students Constitution and Health (CNSSCH), which was conducted every 5 years in 31 provinces of China [25]. The questionnaire had been used for over 20 years before this program and was found to be acceptable for children and their parents. Daily consumption of fruits and vegetables was derived from students’ self-reported frequency (days) and amount (serving, 1 serving = 120 g in raw material) of consumption during the past 7 days. Smoking exposure refers to a combination of objective and passive smoking. Students were asked to choose yes or no to the questions “Have you ever smoked during the past week?” and “Has anybody smoked in your presence during the past week?”, and any “yes” would be counted for the presence of smoking exposure. Daily physical activity included all kinds of outdoor leisure sports (except walking) and was derived from students’ self-reported frequency (days) and time duration (minutes) of outdoor leisure sports during the past 7 days. Information on family history of hypertension was collected from parental questionnaires, using the question “Have you ever been diagnosed by a doctor as hypertension/received medical treatment of hypertension?”. Only a “yes” answer from either father or mother was counted for the presence of family history of hypertension.

### 2.4. Ambient PM_2.5_ Pollutants

Data on daily mean PM_2.5_ levels from 18 January 2013 to 31 December 2013 were obtained from the nearest environmental monitoring station for each selected school. The distance from the school to its nearest monitoring station was between 0.3 km and 5.9 km, with a median of 3.4 km (distances were measured with Google map scale). Annual average of PM_2.5_ concentration was calculated from the daily PM_2.5_ levels.

A 24-h mean of more than 25 μg/m^3^ was regarded as a polluted day with high PM_2.5_ pollution, according to World Health Organization (WHO)’s Air Quality Guidelines (Global Update 2005) [26]. Ratio of polluted days was calculated as days with high PM_2.5_ pollution/days with PM_2.5_ data × 100%.

### 2.5. Statistical Analysis

Descriptive statistics were calculated for all involved variables by quartile groups of annual mean PM_2.5_ concentrations, with continuous variables reported as mean values with standard deviations (SD) and categorical variables reported as numbers with percentages. One-way analysis of variance (ANOVA) and nonparametric test were conducted to evaluate the differences in PM_2.5_ levels.

We conducted two models to assess the association between PM_2.5_ and blood pressure outcomes. In the first model, PM_2.5_ was included as annual means, while in the other model, the ratio of PM_2.5_ polluted days was included. Both indicators were calculated into quartiles, and the blood pressure outcomes of the lowest quartile were set as the reference.

Two-level linear and logistic regression modeling approaches were applied to assess the relationship between PM_2.5_ exposure and SBP, DBP, and HBP, where individuals were treated as the first-level unit and the investigation schools, from where the students were selected, were treated as the second-level unit. Sex, age, BMI, smoking exposure status, daily physical activity time, and family history of hypertension were adjusted as covariates. We also adjusted for daily consumption of fruit and vegetable in sensitivity analyses. Linear trends over the quartiles were tested by trend Chi-square tests.

Based on the results from regression modeling, we further calculated the PAR to estimate the burden of hypertension attributed to long-term exposure to ambient PM_2.5_. The groups with the lowest HBP risk were set as the reference groups, which were the lowest quartile for annual mean categories and the third quartile for the ratio of polluted days categories.

All statistical analyses were performed using Stata 14.0 (StataCorp, College Station, Texas, USA). Results were considered statistically significant at *p* < 0.05 (two-sided).

## 3. Results

**Figure 2:**
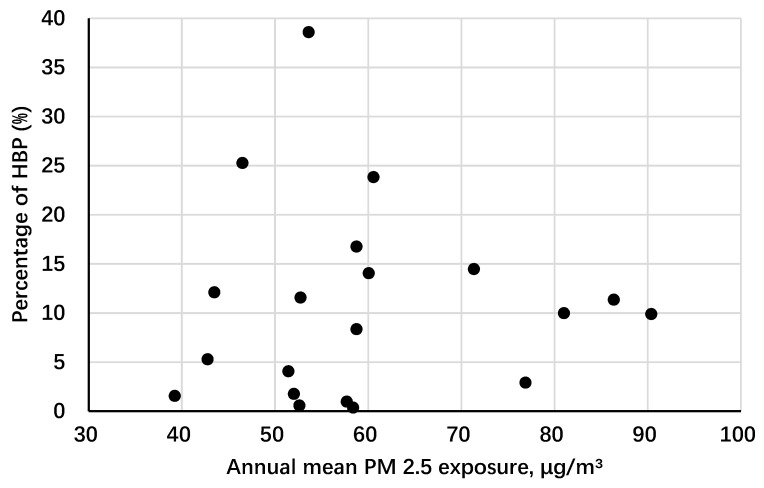
Scatter plot for annual mean PM_2.5_ concentration and high blood pressure prevalence in children from the selected schools. PM_2.5_: particulate matter with an aerodynamic diameter ≤2.5 μg/m^3^.

The descriptive statistics of the study participants are presented in Table 1. The mean age was 10.8 years old (SD: 3.4) and the sex distribution was quite balanced (51.7% boys and 48.3% girls). Among all participants, 9.8% was identified as HBP. Differences between annual mean PM_2.5_ pollution quartiles were all significant. Annual mean concentration of ambient PM_2.5_ pollutants was 63.10 μg/m^3^ (SD: 16.17 μg/m^3^). Schools in Yinchuan were of the lowest exposure level while schools in Tianjin were of the highest level. Figure 2 shows a generally increasing HBP prevalence trend in each selected school over different PM_2.5_ concentrations. 

**Figure 3:**
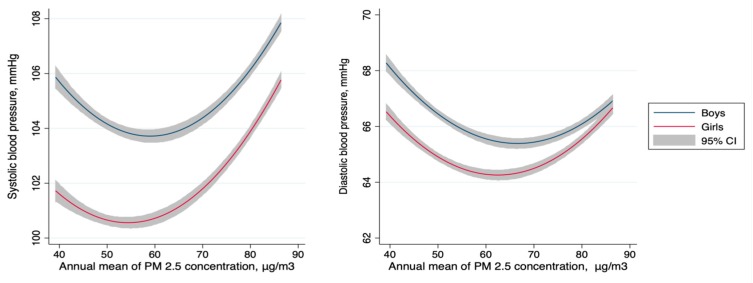
The modeled relationship for annual mean PM_2.5_ concentration and systolic (**left panel**) and diastolic (**right panel**) blood pressure in boys and girls. PM_2.5_: particulate matter with an aerodynamic diameter ≤2.5 μg/m^3^.

As the annual mean of PM_2.5_ increased, a U-shaped association between PM_2.5_ and BP was observed for both SBP and DBP in boys and girls (Figure 3). BP levels slightly decreased along with increasing PM_2.5_ levels below the threshold (57.8 μg/m^3^ for SBP and 65.0 μg/m^3^ for DBP), and then significantly increased along with increasing PM_2.5_ levels above the threshold. This was observed in both SBP and DBP in both sexes. SBP showed more apparent increasing trends above the threshold than DBP in both boys and girls, while boys BP values were higher than girls’, with no more than 4 mmHg.

Table 2 shows quantitative analytic results of BP associated with increasing PM_2.5_ exposure. For HBP and SBP, positive associations were found with increased pollution level and ratio of polluted days. The U-shaped trend was found for DBP especially over the ratio of polluted days categories, and were 0.00 (reference, mmHg), −2.44 (95% CI: −4.90, 0.03), −3.19 (95% CI: −6.04, −0.34), and 0.77 (95% CI: −1.98, 3.52) over quartiles. We also adjusted for daily consumption of fruits and vegetables separately in sensitivity analyses, as these variables represented intakes of dietary antioxidants which may have modification effect. However, adjustment for these variables did not change the results (data not shown). Annual mean concentration of PM_10_, SO_2_, O_3_, NO_2_, CO, air quality index (AQI), annual mean temperature, and humidity were also involved for adjustment (detail of these pollutants was displayed in Table 1), though results were not displayed since no significant differences were observed. PAR was calculated based on the results from logistic regression models, with the quartile with the lowest risk set as the reference group in both quartile categories. In total, PAR was 1.16% (95% CI: 0.80, 1.52) in annual mean categories and 2.85% (95% CI: 2.42, 3.29) in ratio of polluted days categories. As shown in Table 3, PAR was similar in boys and girls, whereas varied over age groups. PAR increased from younger to older age groups for ratio of polluted days.

## 4. Discussion

Results in this study showed that long-term exposure to high level of ambient PM_2.5_ was associated with increased BP level and HBP risk in Chinese children aged 6–18 years. Compared to the reference group with the lowest risk, the PARs were 1.16%–2.85% for HBP in association with higher annual mean concentration and ratio of polluted days. Our study provides evidence that high level of PM_2.5_ pollution may increase the risk of HBP in young generations. The present study was a school-based study and represented a daily situation of PM_2.5_ pollution among general school-aged children, and thus may provide a general view of this issue at a nationwide level.

Studies on the influence of PM_2.5_ on children BP were scarce. Both SBP and DBP were found to have no association with short-term PM_2.5_ pollution in children aged 6–12 years in Belgium [27], whereas DBP was reported to be associated with long-term exposure to PM_2.5_ in the PIAMA cohort study [19,20]. Only one study from Pakistan concluded that both SBP and DBP were positively associated with long-term exposure to PM_2.5_ [21]. In the present study, the association between PM_2.5_ and HBP risk was found to be U-shaped in children, which was similar to what Xie et al. had found in reproductive-age adults in China [28]. Though there was difference between geographical regions and age groups, they found that there was a threshold concentration of 47.9 μg/m^3^ between PM_2.5_ and adulthood hypertension, which was slightly lower than the threshold found in the present study. This trend was hardly reported in other countries, and this may be due to the lack of research data in areas with high air pollution level. We also found a gap of approximately 2–4 mmHg between boys’ and girls’ BP value, which may due to the existence of biological differences between sexes during puberty, since the sex difference P_50_ of SBP and DBP were also in this range. In China, ambient air pollution level during the past decades has increased rapidly with booming urbanization and industrialization. From 2005 to 2014, the average PM_2.5_ concentration in the involved cities had increased more than 70% [29], and the average ratio of polluted days was high up to 44.4% according to the Air Condition Monthly Report of 74 cities in China in December 2015 [30] (Chinese daily PM_2.5_ standard: 75 μg/m^3^). However, more than the fact merely exits in China, this was more likely to be an illustration of the global air pollution situation, as was mentioned in a 2015 updated Global Burden of Disease study that ambient particulate matter was ranked as the sixth most leading risk factor influencing public health worldwide [31]. We assumed from the current results that the influence of PM_2.5_ could be more severe than we expected, and should call for attention globally.

Furthermore, the estimated PAR of HBP risk due to PM_2.5_ concentration ranged from 1.16% to 2.85% in the present study, which was similar to the PAR of 1.7% (35–49 years old) to 2.3% (20–34 years old) found in Chinese reproductive-aged adults [28] but lower than the PAR of 11.75% (5.82–18.53%) found in Chinese people older than 50 [13]. Though there were sex disparities in Chinese adults suggesting that male hypertension was more likely to be attributable to PM_2.5_ [28], we did not obtain similar result from the present study. This might be due to the difference in age groups of the participants. Despite of large amount of confounding factors, we found that the reported PAR from existing studies were lower in reproductive-aged and middle-aged population, and relatively higher in elder population [28,32,33,34,35,36]. In addition, the PAR of HBP associated with high ratio of polluted days was larger than that associated with high annual average concentration in adolescents.

Although there were studies suggesting dietary intake of antioxidants, which are rich in various fruits and vegetables, could modify the adverse effects of PM_2.5_ on BP [37], we did not find the same results in the present study. A possible reason for the difference might be that data of fruit and vegetable consumption were self-reported in students’ questionnaires. To maintain the questionnaire in a proper length for children to complete, we did not use the full-length and detailed food frequency questionnaire, which affects the more precise consumption status. In addition, besides daily consumption, varieties and cooking styles of fruits and vegetables, which were unavailable in the present study, could also affect the intake amounts of antioxidants directly. Lack of information may lead to exposure misclassification and result in null results as reported herein.

Our study has several strengths. First of all, as a large population-based study with wide geographical and age range, the results have good representation for Chinese children. BP was measured twice for each participant according to internationally accepted criteria, and therefore the results were eligible for comparisons with other studies. We analyzed both annual mean concentration and ratio of polluted days and their associations with BP outcomes, providing a more comprehensive view of this topic. In addition, due to the relatively high concentration of PM_2.5_ in these cities, we were able to explore how extremely high level of PM_2.5_ would affect BP in children, which has been hardly investigated in previous population-based studies.

However, there are also several limitations. The study findings were based on a cross-sectional study, and thus we were unable to establish a causal relationship. Because PM_2.5_ data were obtained from fixed monitoring stations near the included schools, it may not reflect the actual exposure to PM_2.5_ for each student at individual level, and potential bias due to exposure misclassification is inevitable. In addition, previous studies have found that the association between PM_2.5_ and BP could be modified by various factors such as traffic noise [38], which we had no access in the present study. Future studies should take a more comprehensive consideration of these confounding factors and conduct further investigation.

## 5. Conclusions

The present study found that the association between long-term exposure to ambient PM_2.5_ and BP outcome appeared to have threshold in Chinese children aged 6–18 years. Both high annual mean concentration and high ratio of polluted days were associated with increased risk of HBP, though further studies revealing causalities should be carried out.

## Figures and Tables

**Table 1:** Characteristics of the participants by annual mean PM_2.5_ pollution categories.

Characteristic	Overall	Quartiles of Annual Mean PM_2.5_ Pollution, μg/m^3^				*P*-Value
		<52.6	52.6–58.9	58.9–81.1	≥81.1	
No. of observations	53,289	16,809	12,629	14,220	9631	
Age, year	10.8 (8.0–13.0)	11.1 (8.0–14.0)	10.6 (8.0–13.0)	10.6 (8.0–13.0)	10.9 (8.0–13.0)	<0.001
Average PM_2.5_, μg/m^3^	63.1 (51.4–81.0)	45.3 (42.8–46.5)	57.3 (57.7–58.8)	73.6 (71.4–81.0)	86.4 (86.4–86.4)	<0.001
Systolic blood pressure, mmHg	103.8 (95.0–110.0)	102.0 (92.5–110.0)	103.3 (94.0–111.0)	103.2 (96.0–110.0)	108.4 (100.0–120.0)	<0.001
Diastolic blood pressure, mmHg	65.9 (60.0–70.0)	65.8 (60.0–71.0)	65.3 (60.0–71.0)	65.1 (60.0–70.0)	68.1 (60.0–70.0)	<0.001
High blood pressure, n (%)	5222 (9.8)	1452 (8.6)	1103 (8.7)	1649 (11.6)	1018 (10.6)	<0.001
BMI, kg/m^2^	18.5 (15.8–20.3)	18.3 (15.8–20.2)	18.3 (15.6–20.3)	18.1 (15.7–20.0)	19.4 (16.0–21.6)	<0.001
Male, n (%)	27,544 (51.7)	8582 (51.1)	6572 (52.0)	7475 (52.6)	4915 (51.0)	0.025
Passive smoking exposure, n (%)	32,804 (61.6)	11,528 (68.6)	7088 (56.1)	8723 (61.3)	5465 (56.7)	<0.001
Family history of hypertension, n (%)	2847 (6.8)	647 (6.2)	930 (8.4)	668 (5.7)	602 (7.1)	<0.001
Daily consumption of fruit, serving	1.3 (0.6–2.0)	1.3 (0.7–2.0)	1.2 (0.6–1.7)	1.2 (0.6–1.7)	1.4 (0.9–2.0)	0.002
Daily consumption of vegetable, serving	1.8 (1.0–2.0)	2.0 (1.0–2.0)	1.7 (1.0–2.0)	1.7 (1.0–2.0)	1.9 (1.0–2.0)	<0.001
Daily physical activity time, minutes	70.1 (25.7–87.9)	67.2 (25.7–85.7)	66.6 (25.7–83.6)	68.4 (24.3–85.7)	84.0 (28.6–98.6)	<0.001
City (No. of environmental monitoring stations near the selected schools)						
Yinchuan (2)	8119 (15.2)	8119 (15.2)	-	-	-	
Shanghai (5)	9059 (17.0)	2861 (5.4)	6198 (11.6)	-	-	
Guangzhou (3)	8601 (16.1)	5829(10.9)	2772 (5.2)	-	-	
Chongqing (3)	10,353 (19.4)	-	3659 (6.9)	6694 (12.5)	-	
Changsha (2)	7526 (14.1)	-	-	7526 (14.1)	-	
Tianjin (2)	9631 (18.1)	-	-	-	9631 (18.1)	

Data are represented as mean (P_25_-P_75_) or number (percentage). PM_2.5_: particulate matter with an aerodynamic diameter ≤2.5 μg/m^3^. BMI: body mass index. *P*-values were calculated with one-way analysis of variance (ANOVA) for continuous variables and Chi-square test for categorical variables.

**Table 2:** Estimated effect on blood pressure associated with ambient PM_2.5_ exposure.

Level of PM_2.5_ Pollution	Effect Estimate (95% CI)		
	HBP (Odds Ratio)	SBP (mmHg)	DBP (mmHg)
Quartile categories for annual mean PM_2.5_, μg/m^3^			
<52.6	1 (Reference)	0 (Reference)	0 (Reference)
52.6–58.9	0.86 (0.33, 2.22)	1.63 (−1.37, 4.62)	−0.11 (−2.72, 2.50)
58.9–81.1	2.24 (0.87, 5.80)	1.27 (−1.72, 4.26)	−0.53 (−3.13, 2.07)
≥81.1	2.13 (0.71, 6.34)	5.59 (2.13, 9.04)	2.60 (−0.41, 5.61)
*P* for trend	0.056	**0.007**	0.246
Quartile categories for ratio of polluted days, % ^a^			
<79.8%	1 (Reference)	0 (Reference)	0 (Reference)
79.9–88.5%	0.65 (0.25, 1.65)	−2.00 (−4.97, 0.96)	−2.58 (−5.03, −0.12)
88.6–93.0%	0.45 (0.15, 1.33)	−1.27 (-4.70, 2.15)	−3.30 (−6.14, −0.47)
≥93.0%	1.24 (0.44, 3.51)	2.54 (−0.76, 5.84)	0.67 (−2.01, 3.39)
*P* for trend	0.868	0.145	0.849

PM_2.5_, particulate matter with aerodynamic diameter ≤2.5 μm; HBP: high blood pressure; SBP: systolic blood pressure; DBP: diastolic blood pressure; CI: confidential interval. ^a^ Indicated to the % of days exceeding the World Health Organization (WHO) 24-h mean PM_2.5_ standard (25 μg/m^3^). Data were adjusted for sex, age, BMI, objective/passive smoking exposure, family history of hypertension, and daily time of physical activity on personal level and school of investigation on the second level. Effect for HBP was odds ratio and 95% confidential interval, effect for SBP and DBP were absolute change in mmHg and 95% confidential interval.

**Table 3:** Estimated high blood pressure burden attributable to ambient PM_2.5_ exposure.

Variable	No. of Observations with HBP, N (%)	Population Attributable Risk % and 95% CI	
		Annual Mean Pollution Level ^a^	Ratio of Polluted Days ^b^
Overall	5222 (9.8)	1.16 (0.80, 1.52)	2.85 (2.42, 3.29)
Sex			
Boys	2913 (10.6)	1.17 (0.81, 1.53)	2.85 (2.41, 3.29)
Girls	2309 (9.0)	1.15 (0.79, 1.51)	2.85 (2.43, 3.28)
Age group			
≤9 years old	2065 (11.5)	1.16 (0.78, 1.54)	2.68 (2.24, 3.12)
10–12 years old	1796 (15.5)	1.20 (0.83, 1.57)	2.75 (2.30, 3.20)
13–15 years old	944 (8.0)	1.12 (0.80, 1.45)	2.93 (2.48, 3.37)
16–18 years old	417 (3.5)	1.16 (0.80, 1.52)	3.14 (2.73, 3.55)

PM_2.5_, particulate matter with aerodynamic diameter ≤2.5 μm; HBP: high blood pressure; OR: odds ratio; CI: confidential interval; PAR: population attributable risk. Groups with the lowest high blood pressure prevalence were set as reference group in calculating population attributable risk (%), that was ^a^ the lowest quartile and ^b^ the third quartile, separately.
